# Radiological considerations in von Hippel-Lindeau disease: imaging findings and the review of the literature

**DOI:** 10.2478/v10019-010-0014-z

**Published:** 2010-09-09

**Authors:** Melda Apaydin, Makbule Varer, Ozgur Oztekin

**Affiliations:** 1 Department of Radiology, Izmir Atatürk Education and Research Hospital, Izmir, Turkey; 2 Department of Radiology, Izmir Education and Research Hospital, Izmir, Turkey

**Keywords:** von Hippel-Lindau, magnetic resonance imaging, brain, spine, tumours

## Abstract

**Background:**

Von Hippel Lindau disease is an autosomal dominant multisystem/multitumoral cancer disease diagnosed by clinical, radiologic and genetic findings. Its prevalence has been estimated to be of 1/36000 inhabitants. The tumours can be benign or malignant.

**Case report:**

We represent MR findings of a family with ten children. Mother and five siblings had von Hippel-Lindau disease.

**Conclusions:**

Radiologic imaging is very important for the early diagnosis and treatment of asymptomatic patients. Diagnosing it early is important because the tumours in von Hippel Lindau disease are treatable. Also, an early detection allows the patient’s survival and quality of life. A multidisciplinary team approach is important in screening.

## Introduction

von Hippel Lindau (VHL) disease was described in von Hippel’s literature in 1911 and Lindau’s literature in 1926.^1^ Symptoms caused by VHL disease depend on the organ which was involved. Patients with the involvement of the central nervous system (CNS) at presentation are usually aged 25–35 years. CNS haemangioblastoma is the most commonly recognized manifestation of VHL disease and occurs in 40% of patients.^2^,^3^ CNS lesions include haemangioblastomas and endolymphatic sac tumours. Visceral manifestations include renal/pancreatic carcinomas and cysts, neuroendocrine tumours and epidydimal cysts. The most important causes of mortality are renal cell carcinoma and cerebellar haemangioblastomas. We represent a family with von Hippel-Lindau disease and discuss imaging findings with regard to the literature.

## Case report

### Patients’ medical history and family history

**FIGURE 1:**
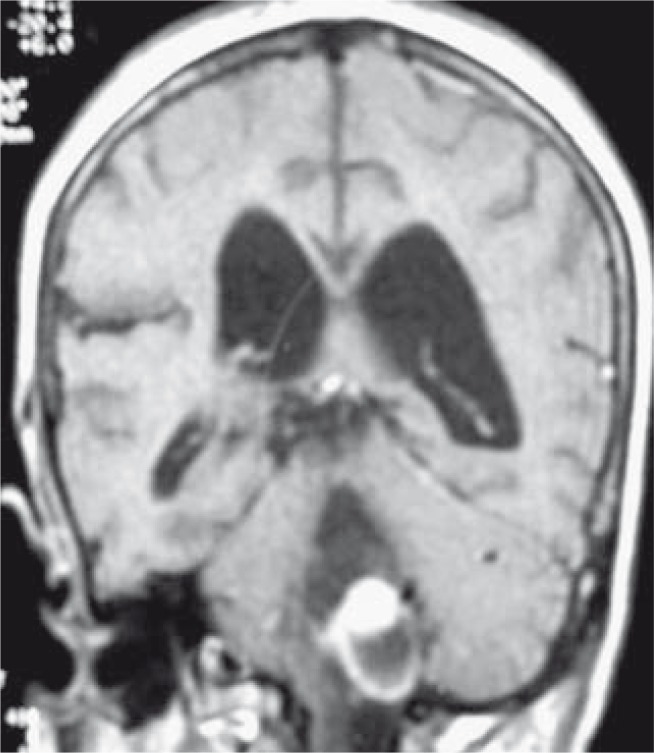
Coronal T1 post-contrast MR image shows a large cystic tumour with enhancing mural nodule in medulla oblongata. There are surgical changes in the *posterior fossa* with dilated fourth and lateral ventricles.

**FIGURE 2:**
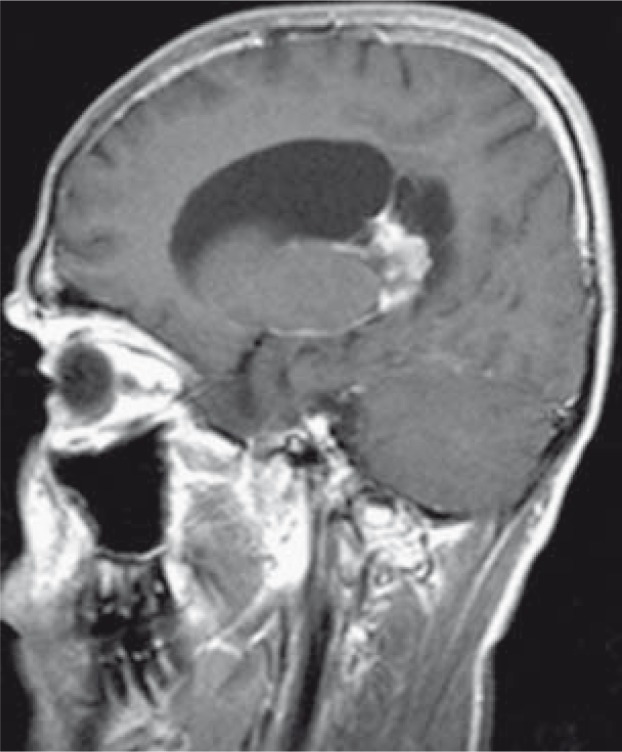
Axial T1 post-contrast MR image shows choroid plexus papilloma in the lateral ventricle.

**FIGURE 3:**
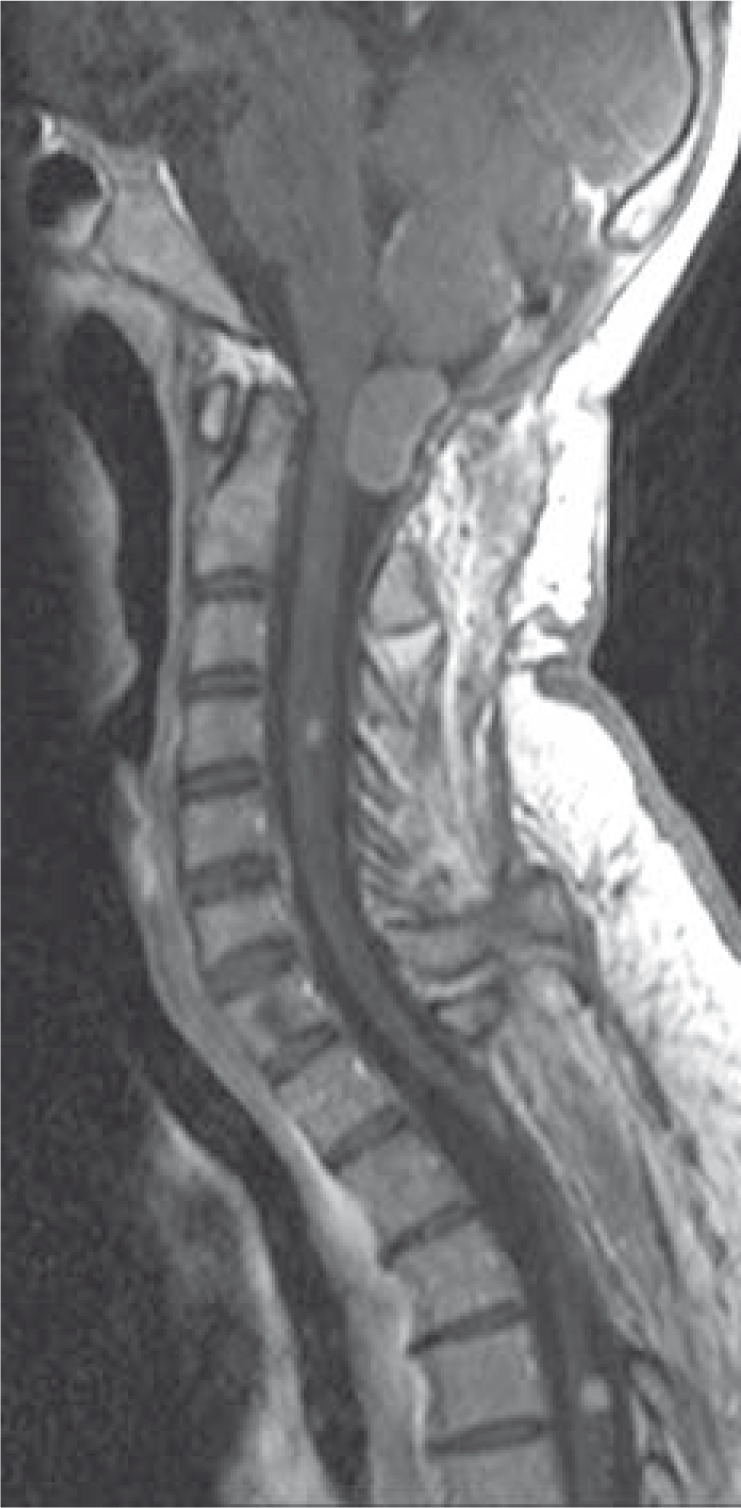
Sagittal T1 post-contrast MR image shows cerebellar haemangioblastoma in the foremen magnum with spinal haemangioblastomas.

**FIGURE 4:**
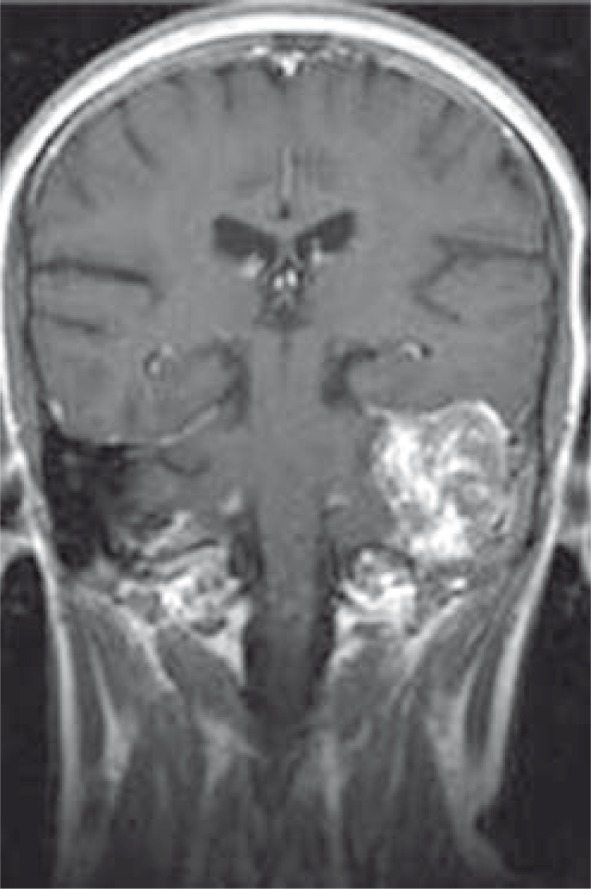
Coronal T1 post-contrast MR image shows endolymphatic sac tumour on the left vestibular aqueduct.

**FIGURE 5:**
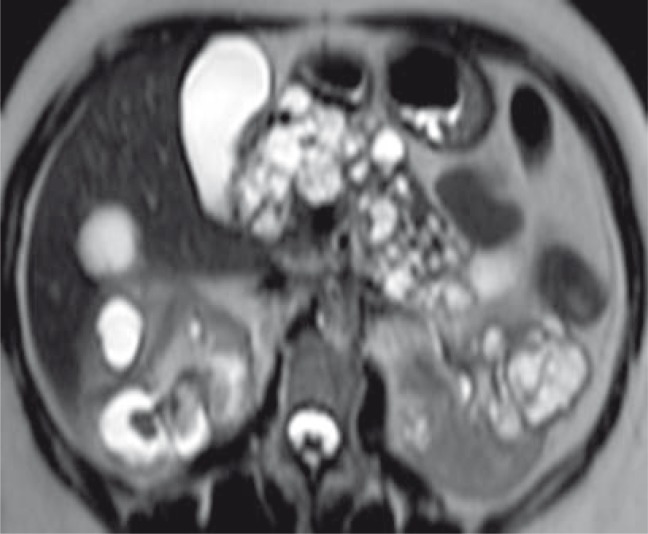
Axial T2 weighted MR image shows multiple cysts in kidney and pancreas.

**FIGURE 6:**
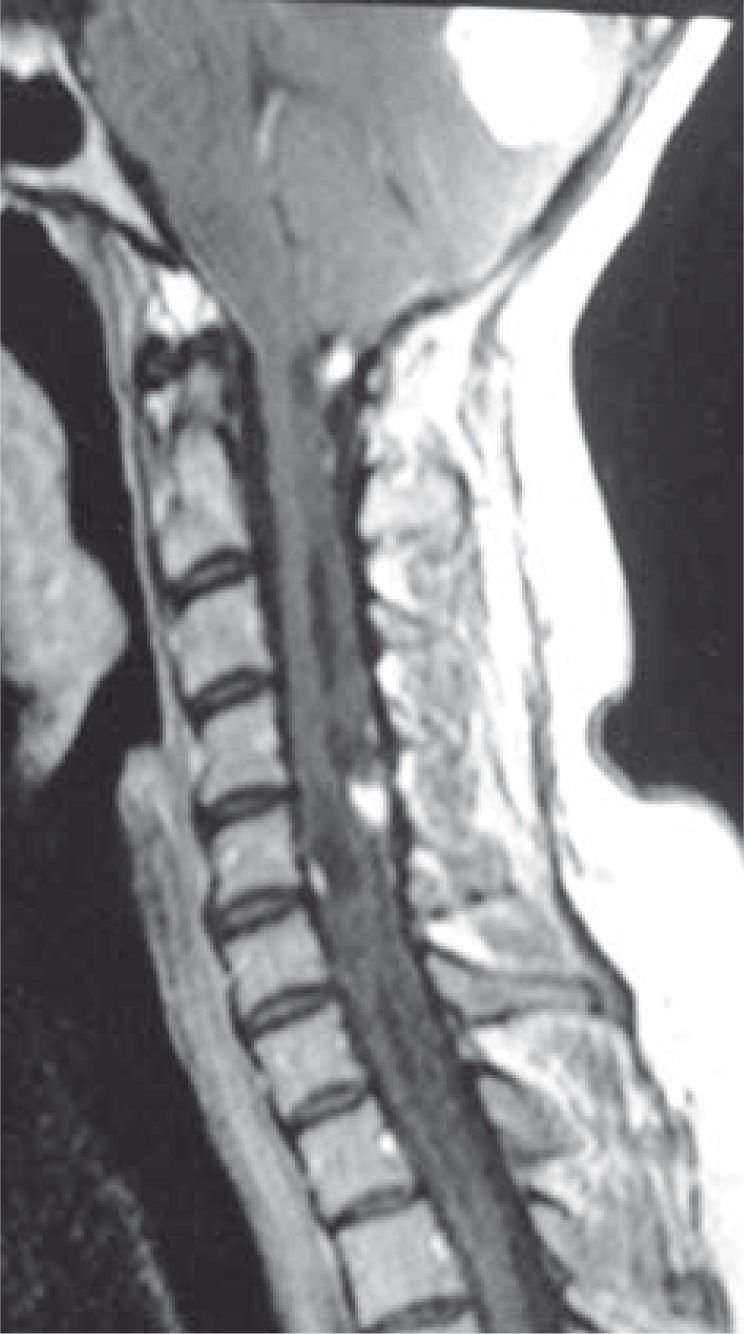
Sagittal Axial T2 weighted MR image shows multiple cysts in kidney and pancreas.

The MR findings of an intermarriaged family with VHL disease were presented. The family had ten children. Two female siblings died from central nervous system haemangioblastomas as like their mother (Figure 1). A 22 year-old male sibling had intraventricular choroid plexus papillomas (Figure 2) with cerebellar and spinal haemangioblastomas (Figure 3). A 33 year-old female patient had headache, tinnitus and abdominal pain. She had a history of endolymphatic sac tumour (Figure 4) treated by surgery. She was found to have cerebellar/spinal haemangioblastomas, pancreas and kidney cystic masses (Figure 5). The younger (21 year-old) asymptomatic female sibling was referred to the radiology department for magnetic resonance (MR) imaging. She was investigated for possible tumours. She was found to have ELST and cerebellar/spinal haemangioblastomas (Figure 6) and underwent radiosurgical ablation (gamma-knife) therapy twice. She has been stabile for three years. The father and the other five siblings were found to be free of the disease.

## Discussion

VHL disease is an autosomal dominant progressive disorder that is associated with various tumours and cysts in the CNS and other visceral organs.^1^–^5^ The VHL gene was identified in 1993 by Latif *et al.* by positional cloning.^2^ The responsible gene is located on the chromosome 3p25–26. The gene has high penentrance but delayed or variable expression and may cause widely different clinical manifestations. VHL disease causes tumours in multiple organs.^4^ Some studies showed that the VHL gene is also inactivated in sporadic renal cell carcinoma, haemangioblastoma and pheochromocytoma.^1^

The clinical manifestation of the disease is reported in 14 different organs with 40 different lesions. These include retinal and CNS haemangioblastomas, endolymphatic sac tumours, renal cell carcinomas and cysts, pancreatic tumours and cysts, pheochromocytomas, and epididymal cystadenomas.^4^,^5^ The most common CNS tumour is haemangioblastoma and occurs in 40% of patients.^3^ Symptoms often begin in the second to third decades of life. Patients may present with neurologic symptoms such as headache, ataxia, and blindness. The exact neurologic deficit depends on the site of the primary lesion.^6^,^7^ The median life expectancy is 49 years.^4^ Usually morbidity and mortality are associated with frequent surgeries to the tumour recurrence. Renal cell carcinomas are the cause of death in 30–50% of the patients.^1^

Molecular genetic testing allows the identification of a deletion or significant mutation that confirms the diagnosis of VHL disease.^4^ But there is also a clinical diagnosis in VHL disease.

The diagnostic criteria for VHL disease are:

- More than 1 haemangioblastoma in the CNS,
- 1 CNS haemangioblastoma and visceral manifestations of VHL, or
- 1 manifestation and a known family history of VHL.^6^

VHL disease was clinically classified into two types. Pheocromocytoma predicts the type. Those which accompanied with this tumour are VHL type 2.^1^

Imaging plays a key role in the identification of abnormalities and in the subsequent follow-up of lesions. It is also important in screening of individuals who are not yet symptomatic.^2^

Recent VHL disease-associated CNS molecular base studies enable new knowledge into their origin and development.^6^ Also the timely diagnosis of this syndrome is important for manifestations.^7^ The high-risk gene carriers must be screened regularly by clinically and radiologically examinations.^4^ One asymptomatic female sibling was found to have endolymphatic sac tumour. She received the therapy before she became symptomatic.

VHL disease can be detected easily by a simple blood test. Accuracy is approaching 100% but the test is not widely available.^5^ A multidisciplinary approach is necessary. Geneticists, neurologists, urologists, gastroenterologists, ophthalmologists, and radiologists are needed to constitute this team.^6^ Screening protocols will vary between centres, but the protocol of National Institute of Health, USA is being widely accepted.^8^ Computed tomography (CT) has the risk of ionizing radiation, which is a problem when screening asymptomatic patients or at-risk relatives.^6^ MRI should be considered instead of CT^5^, because of avoiding ionizing radiation.^9^ CNS manifestations can be detected with great accuracy by MRI.^4^,^6^,^7^ MRI is also effective in the differential diagnosis of the abdominal involvement.^10^–^13^ It is very important not to use gadolinium base MR contrast agents in patients with the renal involvement whose estimated glomerular filtration rate of less than 30 mL/min. There is a risk of *nephrogenic systemic fibrosis.*^14^,^15^ However, *nephrogenic systemic fibrosis* incidence in at-risk patients receiving contrast-enhanced MRI can be reduced after changing contrast administration protocols that include changing the type and dose of the contrast agent.^16^ VHL patients usually have multiple operations for haemangioblastoma. Recently, it is believed that postoperative morbidities are major causes of the physical disability in VHL disease.^6^ The important point is to decide the perfect time for the operation. Mother of the family had died due to haemangioblastoma and hydrocephalus after having multiple operations. Her tumour was located in medulla oblongata. Three siblings had multiple CNS haemangioblastomas. One underwent operation, the other two received stereotactic radiosurgical ablation (gamma knife therapy). This therapy was found to be useful in patients with multiple small haemangioblastomas in VHL disease. Renal cell carcinomas of less than 3 cm in diameter have to be followed by 6 to 12 months period.^4^ One of our patients has multiple cysts in kidney. She has been under MRI follow up in every year with laboratory testing.

There have been important improvements in the management of VHL in the last two decade. The morbidity and mortality of patients with VHL disease has been reduced.^5^ The resection of the tumour, cyst aspiration, stereotactic radiosurgical ablation, photocoagulation, and cryotherapy of any retinal lesions are the choices of the treatment.^4^ VHL is a lifetime disease. Patients need to be constantly checked for the tumours and cysts that develop at various sites in the CNS and visceral organs throughout his/her lifetime. Some patients even receive up to 20 surgical operations in their lifetime to remove tumours.^1^,^17^

The conservative approach to the treatment of VHL lesions is now more widely accepted, a radiologic follow-up with non-invasive imaging especially with MRI is important.
